# Vitamin D receptor gene *BsmI *polymorphisms in Thai patients with systemic lupus erythematosus

**DOI:** 10.1186/ar1910

**Published:** 2006-02-20

**Authors:** Wilaiporn Sakulpipatsin, Oravan Verasertniyom, Kanokrat Nantiruj, Kitti Totemchokchyakarn, Porntawee Lertsrisatit, Suchela Janwityanujit

**Affiliations:** 1 Division of Allergy, Immunology and Rheumatology, Department of Medicine, Faculty of Medicine, Ramathibodi Hospital, Mahidol University, Rama 6 Road, Bangkok10400, Thailand; 2 Research Center, Department of Medicine, Faculty of Medicine, Ramathibodi Hospital, Mahidol University, Rama 6 Road, Bangkok 10400, Thailand

## Abstract

The immunomodulatory role of 1,25-dihydroxyvitamin D3 is well known. An association between vitamin D receptor (VDR) gene *BsmI *polymorphisms and systemic lupus erythematosus (SLE) has been reported. To examine the characteristics of VDR gene *BsmI *polymorphisms in patients with SLE and the relationship of polymorphisms to the susceptibility and clinical manifestations of SLE, VDR genotypings of 101 Thai patients with SLE and 194 healthy controls were performed based on polymerase chain reaction-restriction fragment length polymorphism (PCR-RFLP). The relationship between VDR gene *BsmI *polymorphisms and clinical manifestations of SLE was evaluated. The distribution of VDR genotyping in patients with SLE was 1.9% for BB (non-excisable allele homozygote), 21.78% for Bb (heterozygote), and 76.23% for bb (excisable allele homozygote). The distribution of VDR genotyping in the control group was 1.03% for BB, 15.98% for Bb, and 82.99% for bb. There was no statistically significant difference between the two groups (*p *= 0.357). The allelic distribution of B and b was similar within the groups (*p *= 0.173). The relationship between VDR genotype and clinical manifestation or laboratory profiles of SLE also cannot be statistically demonstrated. In conclusion, we cannot verify any association between VDR gene *BsmI *polymorphism and SLE. A larger study examining other VDR gene polymorphisms is proposed.

## Introduction

The importance of genetic influences on systemic lupus erythematosus (SLE) has been recognized through cumulative genetic epidemiologic studies. Many population-based studies have shown associations between the disease and alleles of immunologically relevant genes, including certain major histocompatibility complex (MHC) loci, Fcγ receptor, and cytokines [1]. 1,25-dihydroxyvitamin D3 is thought to exert many of its action through interaction with a specific intracellular receptor. At the molecular level, 1,25-dihydroxyvitamin D3 inhibits the accumulation of mRNA for interleukin (IL)-2, interferon (IFN)-γ, and granulocyte-macrophage colony-stimulating factor (GM-CSF). At the cellular level, the hormone interferes with T helper cell (Th) function, reducing Th induction of immunoglobulin production by B cells. When given *in vivo*, 1,25-dihydroxyvitamin D3 has been particularly effective in prevention of autoimmune diseases such as experimental autoimmune encephalitis and murine lupus [2]. It has been demonstrated that patients with SLE have a lower level of 25 hydroxyvitamin D3 than do healthy controls [3]. In addition, high-dose 1,25-dihydroxyvitamin D3 and its analog may be useful therapeutic agents for psoriatic arthritis [4] and rheumatoid arthritis [5].

Polymorphism of the vitamin D receptor (VDR) gene was found to be associated with many diseases, including osteoporosis [6], hyperparathyroidism [7], and prostate cancer [8]. An association between VDR gene polymorphism and SLE in Japanese and Chinese patients has been reported with mixed results [9-11]. Although Asians are closely related ethnically, the genetic admixture in Japan or China is different from that of Thailand. Because a high prevalence and high clinical severity of SLE are also observed in the Thai population, we examined the characteristics of VDR gene *BsmI *polymorphisms in a larger cohort of Thai patients with SLE and the relationship of polymorphisms to the susceptibility and clinical manifestations of SLE.

## Materials and methods

This study was conducted in accordance with the principles embodied in the Declaration of Helsinki and was approved by the ethical committees of the Ramathibodi Hospital, Mahidol University, Bangkok, Thailand. DNA from 101 patients with SLE was examined. All patients fulfilled the 1982 revised criteria for SLE [12]. All were females older than 15 years of age. They did not meet criteria for other autoimmune diseases. DNA from 194 unrelated healthy subjects served as controls. All healthy subjects were females older than 15 years of age. VDR genotyping was performed by polymerase chain reaction-restriction fragment length polymorphism (PCR-RFLP).

Genomic DNA was extracted from peripheral white blood cells using standard phenol-chloroform method. PCR was carried out in a final reaction volume of 50 μl. Oligonucleotide primers designed to anneal to exon 7 (primer 1, 5' CAACCAAGACTACAAGTACCGCGTCAGTGA-3') and intron 8 (primer 2, 5'-AACCAGCGGGAAGAGGTCAAGGG-3') were used to amplify 825 bp fragment, including the polymorphic *BsmI *site in intron 7 of the gene. The following reagents were added to a 200-μl ultramicrocentrifuge tube: 5 μl of 10 × buffer (100 mM Tris HCl pH 9.0, 500 mM KCl, and 1.0% Triton x-100), 2 μl of MgCl_2 _(25 mM), 3 μl of deoxynucleotide triphosphate (2 mM each) (Promega, Madison, WI, USA), 0.5 μl of primer 1 (20 μM), 0.5 μl of primer 2 (20 μM), 2.5 units of Taq DNA polymerase (Promega), 300 ng of template DNA, and water to a final volume of 50 μl.

The cycling condition was set as follows: one cycle at 95°C for 3 minutes, 30 cycles at 95°C for 30 seconds, 56°C for 30 seconds, and 72°C for 30 seconds. One final cycle of the extension was performed at 72°C for 10 minutes.

One microliter of the PCR product was digested at 65°C for 1 hour in the final volume of 10 μl with 5 units of restriction enzyme BsmI (New England Biolabs Inc., Ipswich, MA, USA) in 1 × buffer. The digested samples were fractionated by electrophoresis in a 1.5% agarose gel. Restriction fragments were detected by staining with ethidium bromide, and genotypes were determined by comparing the restriction length polymorphism band patterns with a 100 bp DNA ladder run on the same gel. The presence of the BsmI restriction site generated 175 bp and 650 bp fragments, whereas the absence of this site yielded an 825 bp fragment.

The genotypes were classified as excisable allele homozygote (bb), non-excisable allele homozygote (BB), and heterozygote (Bb).

### Statistical analysis

Analyses were performed with Epi Info™ 2002 Results from patients with SLE and control subjects were compared using the χ^2 ^test for statistical significance. Hardy-Weinberg equilibrium (HWE) was determined by Pearson's χ^2 ^goodness-of-fit test.

## Results

The distribution of VDR genotyping in patients with SLE was 1.9% for BB, 21.78% for Bb, and 76.23% for bb. The distribution of VDR genotyping in the control group was 1.03% for BB, 15.98% for Bb, and 82.99% for bb. There was no statistically significance difference between the two groups (*p *= 0.357) (Table 1). The genotype frequencies were consistent with HWE in patients and controls (χ^2 ^= 0.08, *p *= 0.77 and χ^2 ^= 0.14, *p *= 0.71, respectively). The allelic distribution of B and b was similar within the two groups (*p *= 0.173) (Table 2). The relationship between VDR genotype and clinical manifestation or laboratory profiles of SLE cannot be statistically demonstrated (Table 3).

## Dicussion

Most tissues in the body, including heart, stomach, pancreas, bone, skin, gonads, and activated T and B lymphocytes, have the nuclear receptor for 1,25-dihydroxyvitamin D3 (VDR). Thus, it is not surprising that 1,25-dihydroxyvitamin D3 has a multitude of biologic effects that are non-calcemic in nature [13]. Recent research shows that the biologic action of vitamin D extends well beyond the classic function to include effects on immunity, muscle and vasculature, reproduction, and the growth and differentiation of many cell types [14]. 1,25-Dihydroxyvitamin D3 directly inhibits synthesis and secretion of IL-2 [15,16] and IFN-γ [17,18] and also inhibits immunoglobulin production [19]. Genomic actions of 1,25-dihydroxyvitamin D3 are mediated through its nuclear receptor (VDR). The VDR regulates gene transcription by binding to the hexameric core binding motif in promoter region of target genes, VDR element (VDRE) [17]. Extensive studies focused on this VDR gene in various phenotypes have revealed the association between VDR polymorphism and many non-skeletal diseases [20].

Although SLE has features consistent with Th2-type cytokine predominance, both Th1 and Th2 cytokine may be involved in the pathogenesis of SLE [21]. Mononuclear cells of patients with SLE have defects in IL-2 signal transduction and decreased production of IFN-γ [22]. IFN-γ, tumor necrosis factor (TNF)-α, and IL-1 are the most important adhesion molecules inducing cytokine, and they increase in autoimmune renal disease, particularly in Mrl/lpr-Fas and NZB/W mice [23].

Recently, VDR gene *BsmI *polymorphisms have been used as genetic markers to determine their association with SLE [9-11]. A Japanese study of 58 patients with SLE found that the BB genotype might trigger the development of SLE and that the bb genotype was associated with lupus nephritis [9]. A Taiwanese study[10] of 47 Chinese patients with SLE also found an increased distribution of the VDR BB genotype in SLE but indicated no association between the frequency of VDR allelic variations and clinical manifestations or laboratory profiles. In our study, the BB genotype is low in both 194 healthy controls and 101 patients with SLE. However, this is in accordance with previous findings in the Thai population [24]. Thailand is geographically situated in an area between China and India. This genetic admixture may influence the distribution of VDR gene polymorphism. We cannot demonstrate any association between VDR gene *BsmI *polymorphism and SLE. We further examined the relationship between VDR genotype and the individual clinical manifestation or laboratory profiles of SLE, which also cannot be statistically demonstrated.

## Conclusion

It was apparent that compared with the genotype distribution of the VDR gene reported in previous studies [9-11], the genotype frequencies in Thais were different. Because our study includes a larger number of patients and controls than any previous study, we conclude that there is no association between VDR gene *BsmI *polymorphisms and SLE, at least in Thai patients. We propose that other VDR gene polymorphisms be examined.

## Abbreviations

bb = excisable allele homozygote; Bb = heterozygote; BB = non-excisable allele homozygote; HWE = Hardy-Weinberg equilibrium; IFN = interferon; IL = interleukin; SLE = systemic lupus erythematosus; Th = T helper cell; VDR = vitamin D receptor.

## Competing interests

The authors declare that they have no competing interests.

## Authors' contributions

WS conceived of the study, and participated in its design, coordination and acquisition of data. OV carried out the molecular genetic study and performed statistical analysis. KN and KT participated in coordination and interpretation of data. PL participated in acquisition of data and helped in drafting and revising the manuscript. SJ have been involved in drafting and revising the manuscript for important intellectual content and have given final approval of the version to be published. All authors read and approved the final manuscript.

## Figures and Tables

**Table 1:** Distribution of VDR genotyping in patients with SLE and healthy controls

	VDR genotype		
	
	BB	Bb	bb
SLE, *n *= 101 (%)	2 (1.9)	22 (21.78)	77 (76.23)
Control, *n *= 194 (%)	2 (1.03)	31 (15.98)	161 (82.99)

χ^2 ^test = 2.062, *p *= 0.357. Hardy-Weinberg equilibrium test: χ^2 ^= 0.08, *p *= 0.77 in patients and χ^2 ^= 0.14, *p *= 0.71 in controls. bb = excisable allele homozygote; Bb = heterozygote; BB = non-excisable allele homozygote; SLE, systemic lupus erythematosus; VDR, vitamin D receptor.

**Table 2:** VDR allelic frequency in patients with SLE and healthy controls

	B	b
SLE, *n *= 101 (%)	26 (12.87)	176 (87.12)
Control, *n *= 194 (%)	35 (9.02)	353 (90.98)

χ^2 ^test = 2.125, *p *= 0.145. b, excisable allele; B, non-excisable allele; SLE, systemic lupus erythematosus; VDR, vitamin D receptor.

**Table 3:** Relationship between VDR genotype and clinical manifestation or laboratory profiles of SLE

	BB % (ratio) *n *= 2	Bb % (ratio) *n *= 22	bb % (ratio) *n *= 77	Total % (ratio)
Malar rash	50 (1/2)	54.54 (12/22)	54.54 (42/77)	54.45 (55/101)
Discoid rash	0 (0/2)	27.27 (6/22)	31.16 (24/77)	29.70 (30/101)
Photosensitivity	50 (1/2)	31.81 (7/22)	38.96 (30/77)	37.62 (38/101)
Oral ulcer	50 (1/2)	31.81 (7/22)	36.36 (28/77)	35.64 (36/101)
Arthritis	100 (2/2)	77.27 (17/22)	70.12 (54/77)	72.27 (73/101)
Serositis	50 (1/2)	18.18 (4/22)	9.09 (7/77)	11.88 (12/101)
- Pericardial effusion	0 (0/2)	13.63 (3/22)	7.79 (6/77)	8.91 (9/101)
- Pleural effusion	50 (1/2)	9.09 (2/22)	6.49 (5/77)	7.92 (8/101)
Renal disorder	50 (1/2)	68.18 (15/22)	64.93 (50/77)	65.34 (66/101)
Neurologic disorder	0 (0/2)	9.09 (2/22)	20.77(16/77)	17.82 (18/101)
- Seizure	0 (0/2)	9.09 (2/22)	15.58 (12/77)	13.86 (14/101)
- Psychosis	0 (0/2)	0 (0/22)	9.09 (7/77)	6.93 (7/101)
Hematologic disorder				
- Leukopenia	50 (1/2)	40.90 (9/22)	44.15 (34/77)	43.56 (44/101)
- Thrombocytopenia	0 (0/2)	9.09 (2/22)	18.18 (14/77)	15.84 (16/101)
Immunologic disorder				
- Anti-DNA	0 (0/2)	71.42 (15/21)	56.66 (34/60)	60.49 (49/81)
- Anti-Sm	0 (0/2)	57.14 (8/14)	32.60 (15/46)	37.09 (23/62)
ANA	100 (2/2)	100 (22/22)	98.68 (75/76)	99 (99/100)
- Homogenous pattern	50 (1/2)	50 (11/22)	48.68 (37/76)	49 (49/100)
- Rim pattern	0 (0/2)	40.90 (9/22)	44.73 (34/76)	33 (33/100)
- Nucleolar pattern	0 (0/2)	0 (0/22)	3.94 (3/76)	3 (3/100)
- Speckle pattern	50 (1/2)	45.45 (10/22)	59.21 (45/76)	56 (56/100)

ANA = anti-nuclear antibodies; bb = excisable allele homozygote; Bb = heterozygote; BB = non-excisable allele homozygote; SLE, systemic lupus erythematosus; Sm = Smith; VDR, vitamin D receptor.

## References

[B1] Tso BP (2002). The genetics of human Lupus.

[B2] Lemire JM (1992). Immunomodulatory role of 1,25-dihydroxyvitamin D3. J Cell Biochem.

[B3] Muller K, Kriegbaum NJ, Baslund B, Sorensen OH, Thymann M, Bentzen K (1995). Vitamin D3 metabolism in patients with rheumatic diseases: low serum levels of 25-hydroxyvitamin D3 in patients with systemic lupus erythematosus. Clin Rheumatol.

[B4] Huckins D, Felson DT, Holick M (1990). Treatment of psoriatic arthritis with oral 1,25-dihydroxyvitamin D3: a pilot study. Arthritis Rheum.

[B5] Andjelkovic Z, Vojinovic J, Pejnovic N, Popovic M, Dujic A, Mitrovic D, Pavlica L, Stefanovic D (1999). Disease modifying and immunomodulatory effects of high dose 1 alpha (OH) D3 in rheumatoid arthritis patients. Clin Exp Rheumatol.

[B6] Eisman JA (1999). Genetics of osteoporosis. Endocr Rev.

[B7] Carling T, Rastad J, Akerstrom G, Westin G (1998). Vitamin D receptor (VDR) and parathyroid hormone messenger ribonucleic acid levels correspond to polymorphic VDR alleles in human parathyroid tumors. J Clin Endocrinol Metab.

[B8] Ingles SA, Coetzee GA, Ross RK, Henderson BE, Kolonel LN, Crocitto L, Wang W, Haile RW (1998). Association of prostate cancer with vitamin D receptor haplotypes in African-Americans. Cancer Res.

[B9] Ozaki Y, Nomura S, Nagahama M, Yoshimura C, Kagawa H, Fukuhara S (2000). Vitamin-D receptor genotype and renal disorder in Japanese patients with systemic lupus erythematosus. Nephron.

[B10] Huang CM, Wu MC, Wu JY, Tsai FJ (2002). Association of vitamin D receptor gene BsmI polymorphisms in Chinese patients with systemic lupus erythematosus. Lupus.

[B11] Huang CM, Wu MC, Wu JY, Tsai FJ (2002). No association of vitamin D receptor gene start codon fok 1 polymorphisms in Chinese patients with systemic lupus erythematosus. J Rheumatol.

[B12] Tan EM, Cohen AS, Fries JF, Masi AT, McShane DJ, Rothfield NF, Schaller JG, Talal N, Winchester RJ (1982). The 1982 revised criteria for the classification of systemic lupus erythematosus. Arthritis Rheum.

[B13] Holick MF (2004). Vitamin D: importance in the prevention of cancers, type 1 diabetes, heart disease, and osteoporosis. Am J Clin Nutr.

[B14] Malluche HH, Mawad H, Koszewski NJ (2002). Update on vitamin D and its newer analogues: actions and rationale for treatment in chronic renal failure. Kidney Int.

[B15] Lemire JM, Adams JS, Kermani-Arab V, Bakke AC, Sakai R, Jordan SC (1985). 1,25-Dihydroxyvitamin D3 suppresses human T helper/inducer lymphocyte activity *in vitro*. J Immunol.

[B16] Bhalla AK, Amento EP, Krane SM (1986). Differential effects of 1,25-dihydroxyvitamin D3 on human lymphocytes and monocyte/macrophages: inhibition of interleukin-2 and augmentation of interleukin-1 production. Cell Immunol.

[B17] Lemire JM, Archer DC, Beck L, Spiegeburg HL (1995). Immunosuppressive actions of 1, 25-Dihydroxyvitamin D3: Preferential inhibition of Th1 functions. J Nutr.

[B18] Reichel H, Koeffler HP, Tobler A, Norman AW (1987). 1 alpha,25-Dihydroxyvitamin D3 inhibits gamma-interferon synthesis by normal human peripheral blood lymphocytes. Proc Natl Acad Sci USA.

[B19] Iho S, Takahashi T, Kura F, Sugiyama H, Hoshino T (1986). The effect of 1,25-dihydroxyvitamin D3 on *in vitro* immunoglobulin production in human B cells. J Immunol.

[B20] Peacock M, Turner CH, Econs MJ, Foroud T (2002). Genetics of osteoporosis. Endocr Rev.

[B21] Voll RE, Roth EA, Girkontaite I, Fehr H, Herrmann M, Lorenz HM, Kalden JR (1997). Histone-specific Th0 and Th1 clones derived from systemic lupus erythematosus patients induce double-stranded DNA antibody production. Arthritis Rheum.

[B22] Tsokos GC, Boumpas DT, Smith PL, Djeu JY, Balow JE, Rook AH (1986). Deficient gamma-interferon production in patients with systemic lupus erythematosus. Arthritis Rheum.

[B23] Kelley VR, Wuthrich RP (1999). Cytokines in the pathogenesis of systemic lupus erythematosus. Semin Nephrol.

[B24] Ongphiphadhanakul B, Rajatanavin R, Chanprasertyothin S, Chailurkit L, Piaseu N, Teerarungsikul K, Sirisriro R, Komindr S, Puavilai G (1997). Vitamin D receptor gene polymorphism is associated with urinary calcium excretion but not with bone mineral density in postmenopausal women. J Endocrinol Invest.

